# Role of surgery in gynaecological sarcomas

**DOI:** 10.18632/oncotarget.26803

**Published:** 2019-04-02

**Authors:** Valentina Ghirardi, Nicolò Bizzarri, Francesco Guida, Carmine Vascone, Barbara Costantini, Giovanni Scambia, Anna Fagotti

**Affiliations:** ^1^ Division of Gynecologic Oncology, Fondazione Policlinico Universitario Agostino Gemelli, IRCCS, Rome 00168, Italy; ^2^ Catholic University of Sacred Heart, Rome 00168, Italy

**Keywords:** sarcoma, uterine, cervical, ovarian, vulval

## Abstract

Gynaecological sarcomas account for 3-4% of all gynaecological malignancies and have a poorer prognosis compared to gynaecological carcinomas. Pivotal treatment for early-stage uterine sarcoma is represented by total hysterectomy. Whereas oophorectomy provides survival advantage in endometrial stromal sarcoma is still controversial. When the disease is confined to the uterus, systematic pelvic and para-aortic lymphadenectomy is not recommended. Removal of enlarged lymph-nodes is indicated in case of disseminated or recurrent disease, where debulking surgery is considered the standard of care. Fertility sparing surgery for uterine leiomyosarcoma is not supported by strong evidence, whilst available data on fertility sparing treatment for endometrial stromal sarcoma are more promising. For ovarian sarcomas, in the absence of specific data, it is reasonable to adapt recommendations existing for uterine sarcomas, also regarding the role of lymphadenectomy in both early and advanced stage disease. Specific recommendations on cervical sarcomas' surgery are lacking. Existing data on surgical approach vary from radical hysterectomy to fertility-preserving surgery in the form of trachelectomy or wide local excision, however no definite conclusions can be drafted on the recommended surgical approach. For vulval sarcomas, complete surgical excision with at least 2 cm of free margin is considered to be the primary treatment which is associated with good prognosis. The aim of this review is to provide highest quality evidence to guide gynaecologic oncologists throughout surgical management of gynaecological sarcomas.

## INTRODUCTION

Gynaecological sarcomas account for approximately 3% to 4% of all gynaecological malignancies and are associated with poor outcomes compared with gynaecological carcinomas [1]. Uterine sarcomas are approximately 83% of all gynaecological sarcomas. Leiomyosarcoma (uLMS) is the most common histological sub-type, reported in 52% of diagnoses [2], and contributing to a high proportion of death for uterine tumours. For all soft tissue sarcomas, surgery remains the standard of care [3].

In this review, we summarize current available evidences on the role of surgery for uterine, ovarian, cervical and vulval sarcomas in both primary and recurrent setting, to guide surgeons throughout the management of this largely obscure and aggressive disease.

## MATERIALS AND METHODS

The review of the literature included articles published from the inception until May 2018. The search was performed in the Pubmed and Embase databases and included the combination of the following Medical Subjects Heading (MeSH): ‘sarcoma’ & ‘gynecology’, ‘uterine’, ‘cervical’, ‘ovarian’, ‘vulvar’, ‘surgery’, ‘morcellation’. Review articles, books and monographs were also consulted. All pertinent manuscripts were included. Only papers published in English were reviewed. All references were reviewed in order to find other possible manuscripts to be included. The final reference list was generated on the basis of originality and relevance to the broad scope of this review.

## ROLE OF SURGERY IN GYNAECOLOGICAL SARCOMAS

### Uterine sarcomas

Uterine sarcomas are malignant mesenchymal tumours that account for approximately 3% of all uterine malignancies [4]. Current classification includes uLMS, the most common histological subtype (63%), endometrial stromal sarcoma (ESS) (21%) and high-grade or undifferentiated uterine sarcoma (UUS) (16%) [5].

A correct pre-operative diagnosis impacts on all aspects of surgery, from the choice of surgical approach to fertility-sparing surgery.

### Diagnosis

Pre-operative diagnosis of uterine sarcomas remains a challenge. Symptoms may be vague and include uterine bleeding (56%), abdominal distention (52%), pelvic pain or pressure (22%), which are very similar to those presented with benign uterine conditions such as leiomyomas [3].

Moreover, there is no pre-operative test that can reliably diagnose a sarcoma unlike endometrial carcinoma, which can be detected with 90–95% sensitivity by dilation and curettage. Indeed, in a retrospective series of 938 patients with uterine cancer, preoperative sampling was significantly less reliable in predicting the correct histology for uterine sarcomas than for other histological subtypes (64% vs. 81%, *p <* 0.0001) [6].

**Figure 1:**
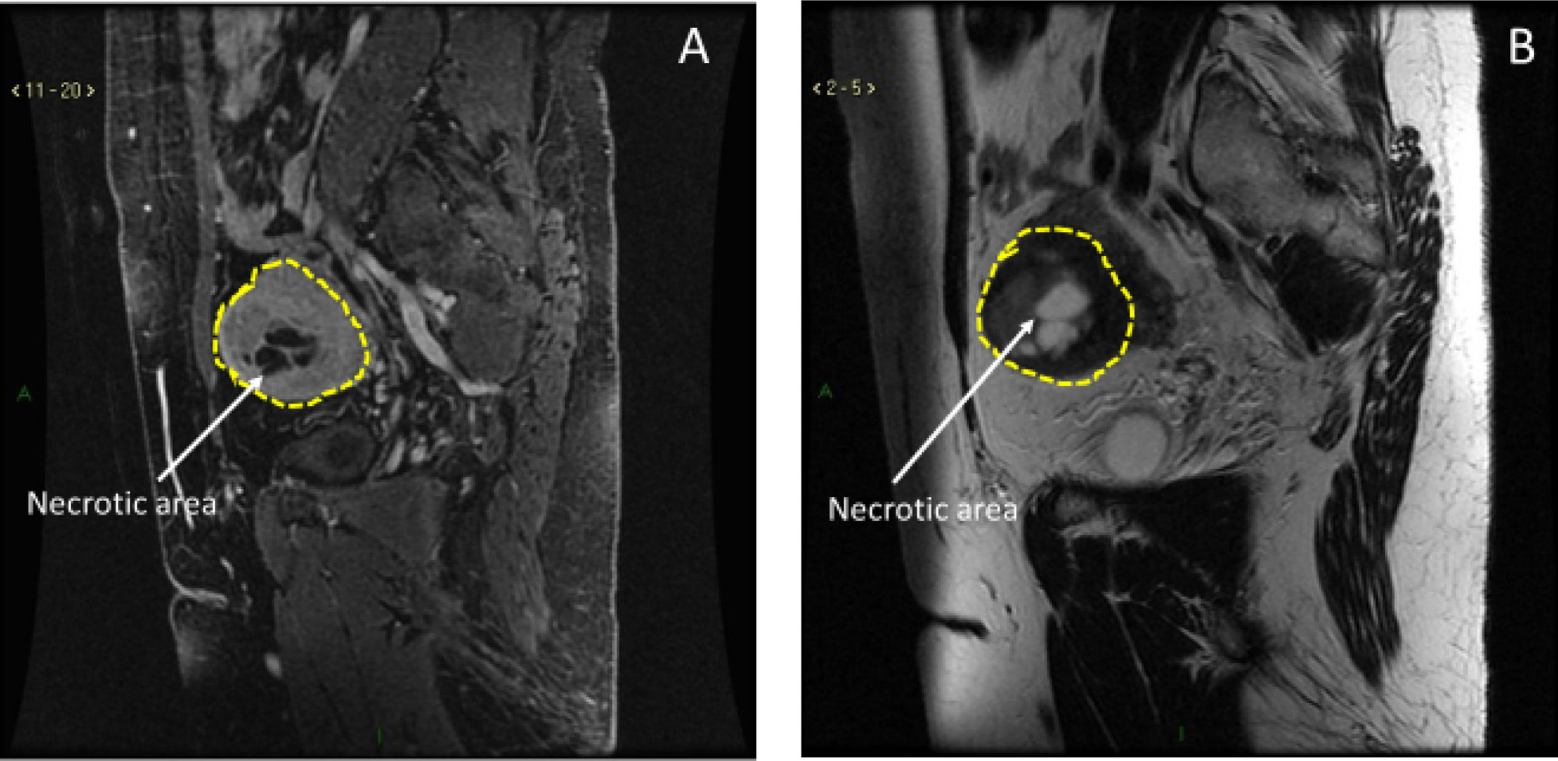
MRI characteristics of uLMS (**A**) Hyperintense signal in T1-weighed images. (**B**) Hyperintense signal in T2-weighed images.

In addition to that, although several features at ultrasound-scan (US-scan) and magnetic resonance imaging (MRI-scan) can raise suspicion of a uterine sarcoma, no pathognomonic images have been identified at any technique [4, 7]. A review of MRI features of uterine sarcomas has been recently published to better define this serious condition, but there is still an overlap in imaging appearance between uLMSs and benign leiomyomas. Uterine sarcomas are more likely to be single lesions, with hyperintense signal on T1 and T2 weighed images (Table 1, Figure 1) [8].

**Table 1:** MRI and US features suggestive for uterine sarcoma [8, 9]

MRI features	US features
Single lesion with irregular margins	Large lesion (>8 cm)
Endometrial thickening	Solid mass
Ascites	Increased central and peripheral vascularity
Hyperintense in T2-weighed images	Degenerative cystic changes
Hyperintense in T1 weighed images	No acustic shadowing

US has been advocated as a promising diagnostic tool for these types of tumours and a lot of effort is made in these days to improve its accuracy. Exacoustos *et al*. have analysed number, size, echotexture, degenerative changes, and vascularity of 32 malignant uterine masses compared to 225 benign leiomyomas showing that uLMS tends to be larger, solitary lesions with increased peripheral and central vascularity [9].

**Figure 2:**
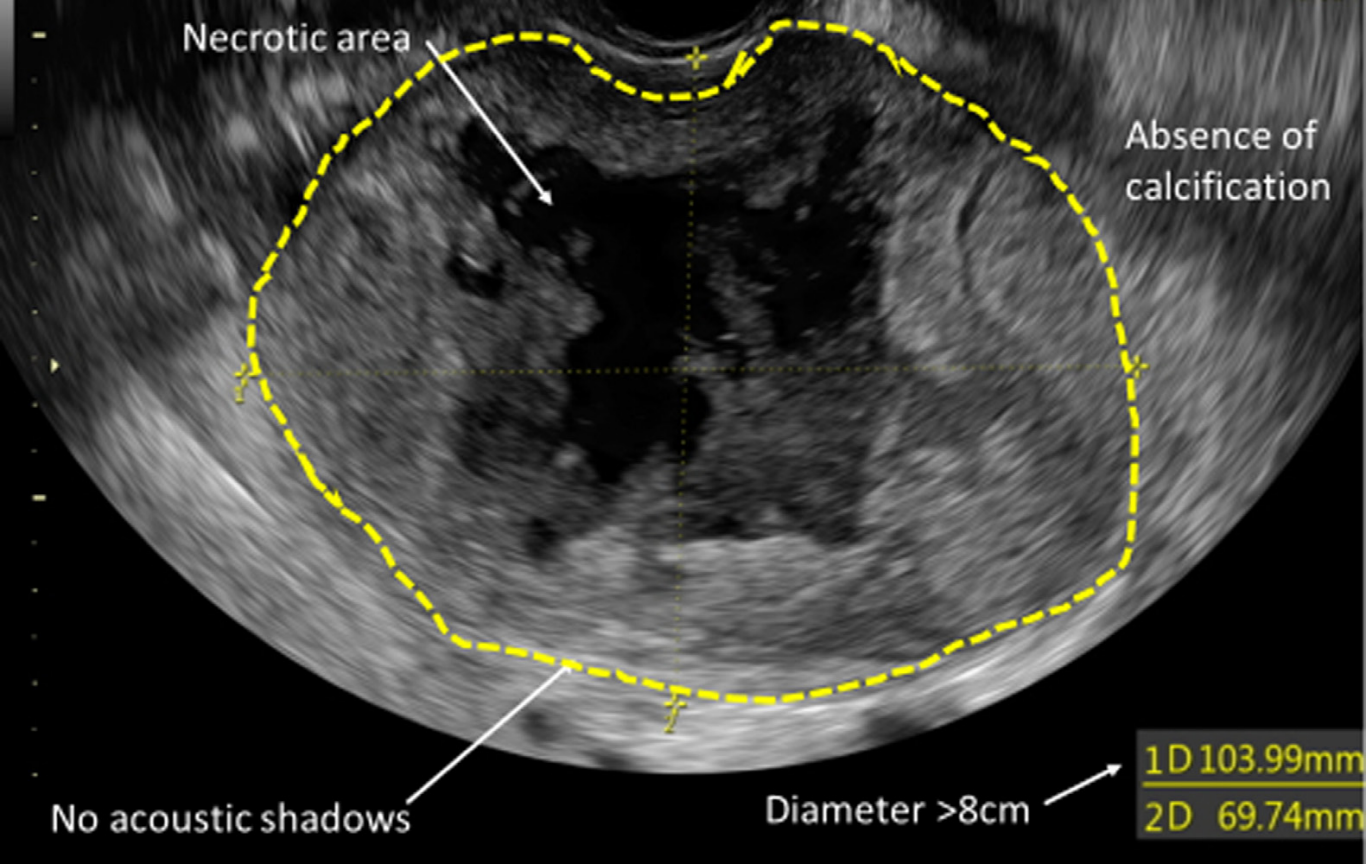
US characteristics of uLMS

Despite this, due to the rarity of these tumours, high quality data on ultrasound characteristics of myometrial masses are lacking and prospective studies are still needed (Table 1, Figure 2).

The value of raised serum lactated dehydrogenase (LDH) as an indicator of malignancy for uterine masses has been advocated. Particularly, together with patient’s older age, it has been found to be a significant predictor for malignancy at multivariate analysis in a retrospective analysis of 63 uterine masses [10].

Although it cannot be considered a diagnostic test, it has been suggested that the combination of degenerative changes within the uterine mass and an increased LDH level, should raise the suspicion of uLMS [11].

As per all soft tissue sarcomas, the role of pre-operative needle biopsy may represent an option either to avoid unnecessary surgeries, or to choose type of surgery in patients with suspicious uterine masses. Kawamura *et al*. [12] investigated the accuracy of needle biopsy for uterine myoma-like tumours in 2002. They performed trans-cervical biopsy in 435 women with uterine masses. Out of them, 7 were confirmed to be uterine sarcomas at final histology and 4 out 7 had a preoperative diagnosis with needle biopsy. They therefore concluded that trans-cervical needle biopsy is a reliable diagnostic test for the differential diagnosis between uterine sarcoma and leiomyoma.

Other more recent evidences have shown sensitivity and specificity of trans-peritoneal biopsy of 92.5% and 100% respectively [13], but no data on survival of patients with biopsied uLMSs is currently available. Indeed, although trans-cervical biopsy may limit the risk of disease spread within peritoneal cavity, it may not reach all uterine masses. On the other hand, trans-peritoneal US-guided biopsy can be performed for all uterine masses, but it raises concerns regarding potential intraperitoneal disease dissemination.

Due to all these diagnostic limitations, a pre-operative diagnosis is quite uncommon. Most women with early stage uterine sarcomas undergo surgery for presumed benign conditions and surgeons have to face a potentially complex surgery with no certainty on diagnosis.

### Surgical treatment of uterine sarcoma confined to the uterus

As per all soft tissue sarcoma, for uterine sarcomas surgery is the standard of care and provides a survival advantage. Resection of disease without fragmentation and with negative surgical margins is the gold standard for treatment [3, 14]. Total hysterectomy (TH) and bilateral salpingo-oophorectomy (BSO) are the standards of care in the management of early stage uterine sarcomas [4].

For both uLMS and ESS, the risk of lymph node metastases has been reported respectively as 3% and <10%, therefore routine lymphadenectomy is not generally recommended in early stage disease (unless suspicious lymphadenopathy is noted on pre-operative imaging or intra-operative findings) to reduce the morbidity related to the procedure [15].

A retrospective evaluation of 1010 ESS patients by Barney *et al*. showed that only age, tumour grade and FIGO stage had a negative impact on survival at multivariate analysis. Adding lymphadenectomy to hysterectomy and BSO did not change survival [16].

The same results are described in a recent retrospective analysis of a large cohort of uLMS patients carried out by Seagle *et al*. where early and complete resection was the best-evidenced treatment for uLMS. Omitting lymphadenectomy was not associated with survival [17].

Regarding the role of oophorectomy in pre-menopausal women with uLMS, data remain unclear. A retrospective review of 1395 leiomyosarcoma patients showed that independent predictors for disease specific survival included age, race, stage, grade, and primary surgery. Oophorectomy did not impact on survival [18]. Overall, since the risk of ovarian metastases has been reported as 4%, ovarian conservation may be considered, without compromising survival outcome, on a case-by-case assessment, only with documented negative endocrine receptor-status [15, 19].

Data on ovarian conservation for ESS are controversial as well. A recent review of 112 patients with low-grade ESS supported the role of oophorectomy to prolong progression-free survival [20].

Uncertainty on this topic is shown in another recent review published by Nasioudis *et al*. They included a cohort of 1482 women affected by uterine sarcoma limited to the uterus. Oophorectomy was not associated with worse oncologic outcomes for women with uLMS, but no conclusions could be made for those with low grade-ESS where ovarian preservation was associated with comparable OS (*p* = 0.410) and cancer specific survival (*p* = 0.560) rates [21]. Also, another series reported no impact on survival for low grade-ESS patients treated with ovarian preservation [22]. Overall, despite further evidences are needed, we can conclude that TH with BSO should be the initial and salvage mainstay treatments for low grade-ESS patients. Ovary-sparing procedures could be considered in young, extensively counselled women, depending on tumour hormonal receptor status; however, long-term follow-up should be mandatory [23]. Regarding high-grade undifferentiated sarcomas, surgery involving TH and BSO is recommended due to the aggressiveness of the disease [24].

### Role of morcellation

With the advent of minimally invasive surgery, laparoscopic morcellation has allowed surgeons to remove large uterine myomas without having to perform open surgery. This has also guaranteed women to experience a reduced number of perioperative complications and a faster recovery [3].

In November 2014, as the risk of morcellating an occult uterine sarcoma could worsen survival outcomes due to potential intra-abdominal disease dissemination, the U.S. Food and Drug Administration (FDA) released a safety communication with a warning regarding the use of the electromechanical morcellator devices for women undergoing myomectomy/hysterectomy [25]. However, some criticisms have been raised with the time at the FDA report. Overall, the risk of morcellating an occult malignancy estimated in the FDA review was 1:352 for any uterine malignancy and 1:498 for uLMS.

Many criticisms have been raised to this report, such as the lack of information regarding the screening process of women undergoing uterine morcellation in the studies included in FDA review [26, 27]. On this topic, a large study was performed at John Hopkins Hospital, which included 2,137 appropriately screened hysterectomy and myomectomy cases. They found only one case in which a woman underwent morcellation of an occult uterine sarcoma over a 10-year period [28].

Because of all the criticisms related to FDA report, several large studies have estimated that the incidence of occult uterine sarcoma in women undergoing surgery for presumed benign fibroids was actually much lower than that quoted by the FDA, on the order of 1 in 1,700 to 7 in 100,000 women undergoing hysterectomy [29, 30].

On the other hand, several studies have put some effort to sustain the FDA communication. In a recent systematic review and meta-analysis of 60 studies, Bogani et. al found that morcellation was an independent negative prognostic factor for survival. It was found to increase the overall (62% vs. 39%; OR: 3.16 (95% CI: 1.38, 7.26)) and intra-abdominal recurrence rate (39% vs. 9%; OR: 4.11 (95% CI: 1.92, 8.81)) as well as the death rate (48% vs. 29%; OR: 2.42) [31]. Superimposable results have been reported in a review by the MITO group, suggesting that morcellation increases the risk of death in patients affected by undiagnosed uLMS [32]. Since uLMS has a high recurrence rate after surgery, the real impact of morcellation on survival is not frankly addressable. However, this procedure may have a more detrimental impact on survival of patients with disease with a lower risk of dissemination, such as low-grade ESS or smooth muscle tumour with uncertain potential (STUMP) [33].

Is therefore true that power morcellation is a dangerous procedure and should be abandoned? Due to the variety of evidences available in literature, a definite conclusion cannot be drafted, and high-quality prospective studies should be undertaken to address this topic. However, it seems useful to underline some bullet points that should be considered in daily practice: i) avoid morcellation when malignancy is suspected; ii) carry out an accurate pre-operative workup to minimise the risk of an undiagnosed occult malignancy [34]; iii) adequate counsel the patient regarding risk to benefit ratio of any proposed procedure. If the decision of a minimally-invasive approach is made and morcellation is an option, contained morcellation into insufflated isolation bags or transvaginal specimen retrieval via endoscopic bags, may represent two promising techniques for a safe specimen extraction.

On this extensive background, practical surgical data on how to behave after an accidental morcellation of a sarcoma need to be provided in this manuscript. Completion hysterectomy or trachelectomy if a supra-cervical hysterectomy has been previously performed, peritoneal biopsies, omentectomy/omental biopsy are advised by multiple investigators. Surgical re-exploration is likely to show findings of disseminated peritoneal sarcomatosis in a significant number of patients diagnosed with uLMS after a morcellation procedure [35, 36]. If that is the case, a cytoreductive procedure is recommended.

### Fertility-sparing surgery

Fertility sparing surgery in uterine sarcomas is an extremely critical subject. In the current literature a limited number of authors have investigated this topic and very few evidences can support this management, which can sometimes be advocated when the sarcoma diagnosis follows a myomectomy in a young and nulliparous patient.

At best of our knowledge, the only series on conservative management for fertility purposes for uLMS was published in 1998 and included 8 patients with a diagnosis of uLMS following myomectomy between 1982 and 1996. Median follow-up was 42 months. 3 pregnancies were recorded and 2 of them had spontaneous delivery at term. The third patient was found to have disseminated disease at the time of caesarean section and died of the disease 26 months after the diagnosis. Basing on their experience, the authors concluded that selected cases of uLMS might be managed conservatively in nulliparous women desiring pregnancy. A strict follow-up is mandatory and at the completion of the reproductive life, a demolitive procedure could be considered [37]. Considering the difficulties in the histological recognition of this disease and the need for histopathological review at referral centres, the different clinical behaviour of these patients may be explained by an incorrect primary diagnosis. Of note, two patients who received a second operation, 24 and 16 months after the first surgery, were found to have benign leiomyomas.

Some successful pregnancies have been reported for ESS conservatively managed; however, data on survival are limited [38, 39].

Overall in literature, 34 cases of ESS conservatively managed are reported and among them, 17 patients (50%) conceived. Fifteen recurrences are documented after a median follow-up of 15 months (range 3–52) with only one death of disease [40].

For all these reasons, it is our opinion that due to the lack of strong evidences and the aggressive nature of those tumours, fertility sparing surgery for uLMS is still an experimental procedure and should not be recommended until more evidences are provided. However, survival data for ESS patients conservatively managed are more reassuring and it can therefore represent an option in selected and extensively counselled nulliparous women.

### Surgical treatment for advanced and recurrent disease

As described by multiple evidences, main treatment for advanced stage uterine sarcoma remains surgery. For uLMS, aggressive cytoreduction seems to be associated with prolonged survival [41], even if in some series, survival improvement after cytoreduction in advanced stage disease is found only for progression-free but not for overall survival. Therefore, selection of surgical candidates needs to be addressed carefully and the improvement in PFS must be weighed against the morbidity of surgery [42].

The same conclusions can be drafted for ESS. A systematic review showed that surgical resection is appropriate for both patients with early-stage (I or II) disease and those with resectable, advanced-stage (III or IV) tumours [43]. Moreover, removal of bulky lymph nodes needs to be considered as part of the cytoreductive procedure, thus inspection is recommended [4].

Few evidences show that surgery may have a role also in the recurrent setting of uterine sarcoma. In a retrospective study performed by Giuntoli *et al*., secondary cytoreductive surgery was found to prolong survival in a selected group of patients with uLMS. Interestingly in their series, neither chemotherapy nor radiation therapy were associated with an improved outcome in this group of patients [44]. The same result was achieved in a more recent Japanese retrospective study, which included 18 women with recurrent uterine sarcoma. They found that secondary cytoreductive surgery led to a survival advantage in this group of patients [45].

Interestingly, the role of hyperthermic intraperitoneal chemotherapy (HIPEC) associated with cytoreductive surgery has been investigated by few studies with good outcomes, whilst evidence on the role of neoadjuvant chemotherapy in this setting are very scanty. A recent retrospective study comparing overall survival of 25 patients with recurrent uterine sarcoma showed survival benefit of the group receiving surgery with subsequent HIPEC compared to surgery followed by chemotherapy or radiotherapy, surgery only or medical treatment alone [46].

Overall, despite all surgical and medical efforts to provide adequate and life-prolonging treatments, prognosis of uterine sarcoma remains quite poor, being recurrence rate after surgery between 50% and 70% and five-year survival rate as low as 30% [47]. It has been shown by multiple evidences that advanced age seems to be associated with unfavourable clinical outcome. Particularly, in a retrospective series of 51 uLMS patients, age older than 50 years old was associated with an 11.07 increased risk of death (*p* = 0.017) at multivariate analysis. [48–50] (Table 2). However, available results on the role of surgery associated with HIPEC seem to be quite promising, therefore further studies on this topic are encouraged.

**Table 2:** Adverse prognostic factor for uterine sarcoma [48, 49]

FIGO stage > II
Age > 50 year old
Tumour size (5-years OS: size <50 mm: 64.0%; 50–100 mm 56.4%; >100 mm 29.3%)
Negative progesterone receptor status
High mitotic count (cut-off > 10 mitoses/10 HPF)
High preoperative CRP serum level (cut-off > 3.5 mg/dL)

HPF: high-power field; CRP: C-reactive protein

Recommendations for surgical management of uLMS and ESS are summarised in Table 3.

**Table 3:** Recommendations for surgical management of uLMS and ESS

Procedure	uLMS	ESS	Level of Evidence
Total hysterectomy	Recommended	Recommended	**IV**
Bilateral salpingo-ophorectomy	Recommended	Recommended	**IV**
Systematic lymphadenectomy in stage I	Not recommended	Not recommeded	**IV**
Debulking surgery in case of disseminated disease	Recommended	Recommended	**IV**
Ovarian preservation in young women	Optionable, if negative ER-PR status	Not recommended	**IV**
Fertility sparing surgery	Not recommended	Optionable in selected patients	**V**

I. Evidence from at least one large randomised, controlled trial of good methodological quality (low potential for bias) or meta-analyses of well conducted randomised trials without heterogeneity

II. Small randomised trials or large randomised trials with a suspicion of bias (lower methodological quality) or meta-analyses of such trials or of trials with demonstrated heterogeneity

III. Prospective cohort studies

IV. Retrospective cohort studies or case–control studies

V. Studies without control group, case reports, experts’ opinions

Supplementary Table 1 shows an overview of main studies on the role of surgery in uterine sarcoma.

### Ovarian sarcomas

Ovarian sarcomas are a very rare entity comprising only 1% of ovarian tumours and available data are limited. These neoplasms have a poorer prognosis compared to epithelial ovarian cancers for all FIGO stages, as shown in a recent retrospective case-control match study [51]. Throughout literature, the most common entities described are ovarian leiomyosarcomas and most of the evidences are in the form of case reports and case series. Primary ovarian leiomyosarcoma (POLMS) is a rare disease with a worse prognosis when compared to their uterine counterpart, diagnosed at the same stage. The prognostic factors that influence overall survival most are tumour stage, size, grade, and mitotic index [51, 52].

These high-risk tumours can arise from smooth muscle of the blood vessels walls, in the cortical stroma, in the corpus luteum, and in the attachment of ovarian ligaments. Interestingly, they can also be of extra-ovarian vascular origin. In particular, primary leiomyosarcomas arising from the ovarian vein have been described as aggressive neoplasms [53].

The diagnostic criteria for ovarian leiomyosarcoma are similar to those used for the uterine counterpart [54].

At best of our knowledge, less than 50 cases of POLMS cases have been reported in the English literature. In all reported cases initial treatment was surgery. The extent of surgery was variable from fertility-preserving operations to complete surgical staging. Adjuvant treatment, either chemo or radiation therapy was administered to many of those cases [51]. Among them, 11 deaths of disease were documented. The longest FU was 118 months.

Of note, POLMS can also arise in the background of a benign ovarian neoplasm. Interestingly, the synchronous presence of a leiomyosarcoma and an ovarian fibroma in a single ovary has been documented in one case whilst in 4 other cases, the leiomyosarcoma was arising in a mature cystic teratoma of the ovary [55, 56].

Regarding surgical treatment of POLMS, up to now there are no prospective studies to define management recommendations for these rare entities. In the absence of specific data, it is reasonable to adapt recommendations from data existing for uLMS. Specifically, for disease limited to the ovary, TH and BSO are recommended. As per uLMS, the likelihood of lymph node metastases or occult malignancy is low, therefore for patients who did not have lymphadenectomy or omentectomy at the time of initial surgery a second operation is not recommended [54]. In the setting of advanced or recurrent disease, reported data are even fewer and, at best of our knowledge, only a small amount of case report is available on this topic. Overall, patients presenting with relapsed disease are candidates for palliative treatment and succumb to their disease within a few months [57]. However, in some cases surgical treatment has demonstrated to prolong survival. Particularly, removal of scalp and liver metastases from POLMS has prolonged survival in two cases [58, 59]. The same result is achieved in a third case with the removal of a large abdominal recurrence 7 months after primary surgery [60].

Ovarian sarcomas include some other less frequent entities such as fibrosarcoma or rhabdomyosarcoma [58], whereas extra-uterine ESS are conventionally referred to as “endometrioid stromal sarcomas” when they arise outside the uterus. For primary endometrioid stromal sarcomas of the ovary scanty data on their behaviour and optimal treatment are available. To date, less than 100 cases of ovarian endometrioid stromal sarcomas have been reported and since most series included both primary and metastatic cases, defining the features of pure ovarian endometrioid stromal sarcomas has been difficult. The largest series of 14 cases has been recently published [61]. The median age at diagnosis was 51.5 years (range 34–61 years), 8 patients underwent TH with BSO, whilst 3 women underwent BSO only because of a previous hysterectomy for benign conditions. The remaining three cases included patients presented with recurrent disease who were initially treated with TH and unilateral salpingo-oophorectomy or BSO only. The median follow-up was 65 months (range 8–311 months). Of the 9 low-grade endometrioid stromal sarcomas cases, 3 developed intraabdominal recurrence at 8–30 and 52 months after surgery with no reported deaths. Of the remaining 5 patients with high-grade endometrioid stromal sarcomas, one was alive and disease free and 4 developed recurrence at 8-12-22 and 24 months after surgery. All patients with recurrent disease were treated with debulking surgery and adjuvant therapy. Of them, 2 succumbed of the disease. Because it may be very challenging to differentiate a primary endometrioid stromal sarcomas from an ovarian metastasis, a thorough analysis of the uterine status is recommended. To conclude, as per other ovarian sarcomas, surgery including TH and BSO is the mainstay of treatment. Tumour debulking should be reserved for advanced stage disease. Despite it does not seem to improve survival, evidences on the role of lymphadenectomy have been elusive [61, 62].

Table 4 presents an overview of main studies on the role of surgery in ovarian sarcoma.

**Table 4:** Overview of main studies on the role of surgery in ovarian sarcoma

Author	Year	Number of patients	Type of study	Setting	Treatment	Survival	Conclusion	Note
Bacalbasa *et al*. [51]	2014	11	Retrospective case control matched study	Prognosis of Ovarian leiomyosarcoma compared to epithelial counterpart	Surgery +CT	FIGO stage II OS 113 months epithelial carcinomas vs 90.5 months for sarcomas (*p <* 0.048); FIGO stage IIIC OS 51months epithelial carcinomas vs 20 months for sarcomas in patients requiring multiple visceral resections. OS 61 months epithelial carcinomas vs 9months for sarcomas in patients not requiring multiple visceral resections. DFS 4.5 months for ovarian sarcomas vs 23.6 epithelial carcinomas. Secondary cytoreduction did not impact on survival (*p <* 0.007); FIGO stage IV OS for sarcoma patients 2 months vs 9 months for epithelial carcinomas (*p <* 0.09)	DFS and OS are lower for patients with ovarian sarcomas compared to epithelial carcinomas	Patients treated from 2002 to 2013
López-Ruiz *et al*. [53]	2017	1	Case report	Primary leiomyosarcoma of ovarian vein	Surgery + CT	Recurred 17 months after surgery (distant metastases)	Primary leiomyosarcomas arising from the ovarian vein are aggressive neoplasms, and the prognosis correlates with stage.	
He *et al*. [55]	2016	1	Case report	Synchronous leiomyosarcoma and fibroma in a single ovary	Surgery + CT	Recurred after 13 months (peritoneal disease)	It is hypothesized that the poor prognosis is associated with the co-occurrence of POLMS and fibroma, which increased the uncertainty of treatment and therapeutic effects	-
Pongsuvareeyakul *et al*. [56]	2017	1	Case report	Leiomyosarcoma and Squamous Cell Carcinoma Arising in Mature Cystic Teratoma of the Ovary	Surgery	Disseminated disease at presentation, died 30 days after surgery for postoperative complications	-	-
Bacalbasa *et al*. [58]	2016	1	Case report	Recurrent disease	Surgery	Recurrence occurred 5 years after diagnosis and was treated with surgery. Alive at 2 years of follow up	-	-
Sultana *et al*. [59]	2009	1	Case report	Recurrent disease	Surgery + CT	Recurred 18 months after diagnosis. No data on follow up	-	-
Rasmussen *et al*. [60]	1997	1	Case report	Recurrent disease	Surgery + CT at primary diagnosis. Surgery only at recurrence.	First recurrence after 41 months. Second recurrence 70 months after secondary cytoreduction. Alive at 7 years of follow up		
Xie *et al*. [61]	2017	14	Retrospective study	Clinicopathologic and outcome of primary ovarian ESS patients	Surgery: 14 CT: 10 RT: 2 HT: 3	At a median follow up of 65 months (range 8–311 months): All 9 low grade ESS patients were alive 3 (33.3%) recurred after surgery Among 5 high grade ESS patients: 1 (20%) did not recur and was alive 4 (80%) recurred 2 (40%) died of disease	-	-
Geas *et al*. [62]	2004	1	Case report	Primary ESS arising from endometriosis	Surgery + HT	No evidence of recurrence at follow up	-	-

^*^ESS: endometrioid stromal sarcoma; ^*^FU: follow up; ^*^CT: chemotherapy; ^*^RT: radiotherapy; ^*^HT: hormonal therapy. DFS: disease free survival; OS: overall survival; CT: chemotherapy; POLMS: primary ovarian leiomyosarcoma; ESS: endometrioid stromal sarcoma; RT: radiotherapy; HT: hormonal therapy

### Cervical sarcomas

Cervical sarcomas account for less than 1% of all cervical malignancies. Most of the patients present with vaginal bleeding and a bulky cervical mass at the time of diagnosis [63].

Due to the relative infrequency of the disease, most of the available data on the natural history of cervical sarcomas are derived from case reports and small case series, thus leading to paucity of information regarding the clinical features, treatment modality and prognosis of patients [64]. The largest available series of cervical sarcomas identified 323 cases among 33,074 patients with cervical cancer treated over nearly 17 years. Compared to women with squamous cell carcinoma and adenocarcinoma, patients with cervical sarcomas tended to be younger, have larger tumours, and have more advanced stage disease with worse prognosis with respect to the epithelial counterpart matched by stage [65].

Among all the subtypes, rhabdomyosarcoma of embryonal subtype is the most frequently reported in young patients. In a retrospective review of 11 patients by Kriseman *et al*., information regarding this rare histological entity were collected from 1980 to 2010 at MD Anderson Cancer Centre. All patients underwent surgery at some stage of their treatment, 9 as upfront surgery and 2 after medical treatment. In their series, 1 patient died for the disease and 1 died for complication related to chemotherapy [66].

Overall, the prognosis associated with cervical rhabdomyosarcoma appears to be favourable. In particular, classical embryonal rhabdomyosarcoma, most commonly presenting with a polypoid (exophytic) growth pattern, is associated with better prognosis. Among the embryonal subtypes, the botryoid variant is associated with better outcomes [67].

Since such a few evidences on this topic are available in literature, it is difficult to draw broad conclusions on treatment modality. However, it appears that surgery and chemotherapy are the mainstays of treatment of cervical rhabdomyosarcoma. The surgical approaches described in literature for these extremely uncommon entities vary from radical hysterectomy to fertility preserving surgery in the form of radical/simple trachelectomy or wide local excisions. However, the paucity of cases described, and the lack of survival data do not allow to identify the recommended surgical approach [68, 69]. For the same reason, no definite answer can either be given on impact of nodal metastases on survival and therefore on the role of lymphadenectomy [70].

Leiomyosarcomas arising in the uterine cervix are exceedingly rare tumours. They tend to arise in the perimenopausal period with abnormal vaginal bleeding as the most common presenting symptom.

Due to limited evidences, we must accept means of managing uLMS for guidance, i.e. TH and BSO. The role of lymphadenectomy is limited due to the low rate of lymphatic spread. Metastases are more likely to be found when the nodes are grossly enlarged or in the setting of obvious intra-abdominal disease [70].

Despite a thorough literature search on this topic, all available evidences appear not to be up to date.

Table 5 shows an overview of main studies on the role of surgery in cervical sarcoma.

**Table 5:** Overview of main studies on the role of surgery in cervical sarcoma

Author	Year	Number of patients	Type of study	Setting	Treatment	Survival	Conclusion	Note
Khosla *et al*. [64]	2012	8	Retrospective	Primary disease	Primary surgery in 3 patients ± CT ± RT Primary RT 2 in 2 patients 3 patients absconded after diagnosis	3 patients (37.3%) alive without disease. 2 (25%) disease related death. 3 (37.5%) lost at FU	The optimal management of these tumors is uncertain owing to its rarity; however, combined modality treatment can result in prolonged survival.	Patients treated from 2006 to 2009
Bansal *et al*. [65]	2010	323	Retrospective	Primary disease. Comparison of outcome with adenocarcinoma and squamous cell cervical cancer	NR	5 years OS: FIGO Stage IA 95% (95% CI, 94–95%) for squamous neoplasms vs 80% (95%, CI 39–95%) for sarcomas; FIGO stage IB 80% (95%, CI 79–81%) for squamous neoplasms vs 67% (95%, CI 58–75%) for sarcomas; FIGO stage III 32% (95%, CI 30–34%) for squamous neoplasms vs 20% (95%, CI 7–39%) for sarcomas	The prognosis for women with cervical sarcomas is inferior to that of squamous cell and adenocarcinomas matched by stage.	National Cancer Institute’s Surveillance, Epidemiology, and End Results Program (1988–2005)
Kriseman *et al*. [66]	2012	11	Retrospective	Primary disease. Cervical rhabdomyosarcoma	Surgery in 9 patients, CT in 2 patients	At a median follow-up of 23 months (range, 1–176 months), 3 patients (27%) recurred. 1 patient died for chemotherapy related complications after recurrence. At last FU 1 patient who recurred died of disease. Of the 8 patients who did not recur, 2 deaths were recorded (1 for unknown cause and 1 for a different cancer), 6 patients were alive without disease	Surgery and chemotherapy are the mainstays of treatment of cervical rhabdomyosarcoma, and the prognosis of patients treated with multimodal therapy is good	Patients treated from 1980–2010
Ditto *et al*. [68]	2013	1	Case report	Primary disease. Cervical rhabdomyosarcoma	Surgery + CT + RT	No evidence of recurrence at a follow up of 46 months	-	-
Li *et al*. [69]	2011	3	Retrospective	Primary disease. Radical abdominal trachelectomy in cervical malignancies	Surgery	No recurrence at median follow up of 22.8 months (range 1–78 months)	The surgical, oncological and fertility outcomes of this study suggested radical abdominal trachelectomy as an appropriate management for cervical malignancies	Patients treated from 2004 to 2010
Irvin *et al*. [70]	2003	1	Case report	Cervical leiomyosarcoma	Surgery + RT	At 5 years follow up No evidence of disease	-	-

CT: chemotherapy; RT: radiotherapy; NR: not reported; OS: overall survival

### Vulval sarcomas

Vulval sarcomas account for 1–3% of all vulval cancers. The most common vulval sarcomas are leiomyosarcomas, followed by other less frequent histologic types, like angiosarcomas, malignant peripheral nerve sheath tumours, and malignant fibrous histiocytomas [71, 72].

Vulval rhabdomyosarcoma is more frequent in childhood and adolescence and comprises more than a half of soft tissue sarcomas in paediatric patients. The adult variant is associated with a poorer prognosis [73].

All of these tumours are characterised by no-specific symptoms and are often confused with benign conditions, such as Bartolin’s cyst or vulval abscesses. Symptoms may include chronic vulval pruritus, vulval mass or longstanding pain. [74].

The prognosis of these neoplasms is generally poor. They tend to have rapid growth, high metastatic potential, frequent recurrences, aggressive behaviour, and high mortality rate [71].

For diagnostic purposes, tumours showing three or all of the four following features should be considered as sarcomas: ≥5 cm in greatest dimension, infiltrative margins, ≥5 mitotic figures per 10 high power field, and moderate to severe cytologic atypia [75].

Most of the available literature on management of vulval sarcomas is based on case reports and, as per all gynaecological sarcomas, evidences are lacking, and no definite algorithms are available.

However, complete surgical excision with negative margins is considered to be the primary treatment, which is associated with good prognosis. For residual tumour, combined chemotherapy and radiotherapy is advocated [75, 76].

This data is also confirmed by a series of 24 women with vulval (9 patients) and vaginal (15 patients) sarcoma published by Curtin *et al*. [77]. They performed surgical excision with free margins as primary treatment in 23 of them, without adjuvant therapy. Among the vulval sarcoma cases, only one out of 7 patients with low-grade leiomyosarcoma recurred locally after this treatment. Basing on their experience, surgery should be considered the primary therapy for those neoplasms, whereas adjuvant treatment is indicated in high-grade tumours or locally recurrent low-grade sarcomas.

In a retrospective study performed by Aarsten *et al*., no difference was found in the biological behaviour of vulval sarcomas, when compared to sarcomas originating in other parts of the body. For all histologic types excepting epithelioid sarcomas, wide surgical excision was associated with improved prognosis. Moreover, neither elective groin node dissection, nor excision of enlarged inguinal lymph nodes was beneficial in most of the cases [76].

As mentioned before, vulval epitheliod sarcoma is considered to have a high rate of local relapses, regional nodal spread and distant metastases. For this aggressive disease, the role of apparently negative groin lymph node dissection is still debated [78]. In the same way, evidences on removal of enlarged groin nodes are controversial [79].

Overall, standard management of vulval sarcomas is still not well established due to their rarity. However, surgery with free margins is the cornerstone of treatment. The width of the margin is still not fully defined but, basing on data from extragenital location, 2 cm is considered sufficient [80]. If systematic groin node dissection or removal of enlarged groin nodes has a benefit on survival is still debated and further evidences are needed. The role of adjuvant treatment has been advocated for patients with close surgical margins, residual tumour or high-grade disease [80].

### Vaginal sarcomas

Vaginal sarcomas are infrequent entities which can also occur in pediatric patients. Different histotypes are described in literature, although very limited data are available due to their rarity. In a retrospective review of 144 cases of rhabdomyosarcoma of lower female genital tract, Nasioudis *et al* identified 74 women (54.4%) with vaginal or vulval rhabdomyosarcoma with an overall patient’s median age of 16 years (range, 1–87) [73]. Embryonal rhabdomyosarcoma is the most common subtype, for which radical surgery has been mostly replaced with chemotherapy, radiation treatment and conservative surgery. On this topic, a case of vaginoscopic resection of vaginal rhabdomyosarcoma during infancy has been reported [81]. Among paediatric tumours, 11 cases of extra-osseus primary Ewing sarcoma of the vagina have been described in literature. For those patients, surgical treatment is an option only in absence of distant metastases [82].

ESS rarely occur outside the uterus and even more rarely they arise in the vagina. At best of our knowledge, only seven cases have been described not in association with endometriosis. Due to the infrequency of the disease, there are not specific guidelines to follow and for this reason treatment is mainly based on previous case reports and treatment guidelines of ESS [83].

Feasibility of laparoscopic surgery in this setting in opposition to laparotomy has been recently described by Pontrelli *et al* [84], who reported no recurrence at a follow up of 29 months after complete laparoscopic resection of a vaginal adenosarcoma.

Table 6 shows an overview of main studies on the role of surgery in vulval and vaginal sarcomas.

**Table 6:** Overview of main studies on the role of surgery in vulval and vaginal sarcomas

Author	Year	Number of patients	Type of study	Setting	Treatment	Survival	Conclusion	Note
Nasioudis *et al.* [73]	2017	144	Retrospective	Rhabdomyosarcoma of lower genital tract	Surgery + adjuvant CT	5-year OS 68.4%. Median OS for women with stage IV RMS was 8 months (95% CI 0, 26.6)	Prepubertal and adolescent age display greater survival rates. Older age, advanced stage disease and non-embryonal histologic subtypes are associated with inferior outcomes.	National Cancer Institute’s Surveillance, Epidemiology, and End Results Program (1973–2013)
González-Bugatto *et al.* [74]	2009	1	Case report	Vulval leiomyosarcoma arising in Bartholin’s gland	Surgical excision	Recurrence 12 months after surgery. Alive after 5 years of follow up	-	-
Curtin *et al.* [77]	1995	24	Retrospective	Primary disease. Vaginal and vulval sarcomas	Surgery in 22 patients	16 patients were (70%) are free of disease at a median follow-up time of 47 months (range 12–156, mean 59). Five women died of progressive disease and two were alive al last follow up with persistent or recurrent disease	-	Patients treated from 1974 to 1993
Kim *et al.* [79]	2008	1	Case report	Primary disease. Vulval epithelioid sarcoma	Wide local excision	Alive at 8 months follow up	-	-
Ulutin et al. [80]	2003	7	Retrospective	Primary disease. Vulval soft tissue sarcoma	Surgery ± groin lymph node dissection ± adjuvant RT	No recurrence after a median follow up of 127.8 months	-	Data from Sidney Kimmel Comprehensive Cancer tumour registry. Patients treated from 1977 to 1997
Ghada *et al.* [83]	2017	1	Case report	Primary ESS arising in the vagina	Surgery + hormonal treatment	No recurrence after 7 months of follow up	-	-

OS: overall survival; CT: chemotherapy; RT: radiotherapy; ESS: endometrial stromal sarcoma

## CONCLUSIONS

Gynaecological sarcomas represent a wide spectrum of neoplasms that, due to their rarity, are still partially obscure. For all of them, surgical treatment is the cornerstone of care providing survival advantage, despite the prognosis is poor. Other additional treatment strategies such as chemotherapy and radiotherapy are available for these patients, although their role has not been investigated in this review. Prospective data are needed to better characterise these uncommon entities and to potentially standardise treatment modalities worldwide.

## SUPPLEMENTARY MATERIALS TABLE




