# Supplementary material for: Role of surgery in gynaecological sarcomas
Source: Oncotarget. 2019 Apr 2;10(26):2561–75. doi: 10.18632/oncotarget.26803 (PMC6493462; doi:10.18632/oncotarget.26803)
Supplement: Supplementary file 1 [file oncotarget-10-2561-s001.pdf]

Role of surgery in gynaecological sarcomas

SUPPLEMENTARY MATERIALS

Supplementary Table 1: Overview of main studies on the role of surgery in uterine sarcoma. See Supplementary Table 1

REFERENCES

1. Barney B, Tward JD, Skidmore T, Gaffney DK. Does radiotherapy or lymphadenectomy improve survival in endometrial stromal sarcoma? Int J Gynecol Cancer. 2009; 19:1232–38. https://doi.org/10.1111/IGC.0b013e3181b33c9a.
2. Seagle BL, Sobecki-Rausch J, Strohl AE, Shilpi A, Grace A, Shahabi S. Prognosis and treatment of uterine leiomyosarcoma: A National Cancer Database study. Gynecol Oncol. 2017; 145:61–70. https://doi.org/10.1016/j.ygyno.2017.02.012.
3. Kapp DS, Shin JY, Chan JK. Prognostic factors and survival in 1396 patients with uterine leiomyosarcomas: emphasis on impact of lymphadenectomy and oophorectomy. Cancer. 2008; 112:820–30. https://doi.org/10.1002/cncr.23245.
4. Leitao MM, Sonoda Y, Brennan MF, Barakat RR, Chi DS. Incidence of lymph node and ovarian metastases in leiomyosarcoma of the uterus. Gynecol Oncol. 2003; 91:209–12. https://doi.org/10.1016/S0090-8258(03)00478-5.
5. Stewart LE, Beck TL, Giannakopoulos NV, Rendi MH, Isacson C, Goff BA. Impact of oophorectomy and hormone suppression in low grade endometrial stromal sarcoma: A multicenter review. Gynecol Oncol. 2018; 149:297–300. https://doi.org/10.1016/j.ygyno.2018.03.008.
6. Nasioudis D, Chapman-Davis E, Frey M, Holcomb K. Safety of ovarian preservation in premenopausal women with stage I uterine sarcoma. J Gynecol Oncol. 2017; 28:e46. https://doi.org/10.3802/jgo.2017.28.e46.
7. Shah JP, Bryant CS, Kumar S, Ali-Fehmi R, Malone JM Jr, Morris RT. Lymphadenectomy and ovarian preservation in low-grade endometrial stromal sarcoma. Obstet Gynecol. 2008; 112:1102–08. https://doi.org/10.1097/AOG.0b013e31818aa89a.
8. Bai H, Yang J, Cao D, Huang H, Xiang Y, Wu M, Cui Q, Chen J, Lang J, Shen K. Ovary and uterus-sparing procedures for low-grade endometrial stromal sarcoma: a retrospective study of 153 cases. Gynecol Oncol. 2014; 132:654–60. https://doi.org/10.1016/j.ygyno.2013.12.032.
9. Lissoni A, Cormio G, Bonazzi C, Perego P, Lomonico S, Gabriele A, Bratina G. Fertility-sparing surgery in uterine leiomyosarcoma. Gynecol Oncol. 1998; 70:348–50. https://doi.org/10.1006/gyno.1998.5124.
10. Xie W, Cao D, Yang J, Jiang X, Shen K, Pan L, Huang H, Lang J, You Y, Chen J. Fertility-sparing surgery for patients with low-grade endometrial stromal sarcoma. Oncotarget. 2017; 8:10602–08. https://doi.org/10.18632/oncotarget.12491.
11. Dinh TA, Oliva EA, Fuller AF Jr, Lee H, Goodman A. The treatment of uterine leiomyosarcoma. Results from a 10-year experience (1990-1999) at the Massachusetts General Hospital. Gynecol Oncol. 2004; 92:648–52. https://doi.org/10.1016/j.ygyno.2003.10.044.
12. Leitao MM Jr, Zivanovic O, Chi DS, Hensley ML, O'Cearbhaill R, Soslow RA, Barakat RR. Surgical cytoreduction in patients with metastatic uterine leiomyosarcoma at the time of initial diagnosis. Gynecol Oncol. 2012; 125:409–13. https://doi.org/10.1016/j.ygyno.2012.02.014.
13. Giuntoli RL 2nd, Garrett-Mayer E, Bristow RE, Gostout BS. Secondary cytoreduction in the management of recurrent uterine leiomyosarcoma. Gynecol Oncol. 2007; 106:82–88. https://doi.org/10.1016/j.ygyno.2007.02.031.
14. Nakamura K, Kajiyama H, Utsumi F, Suzuki S, Niimi K, Sekiya R, Sakata J, Yamamoto E, Shibata K, Kikkawa F. Secondary cytoreductive surgery potentially improves the oncological outcomes of patients with recurrent uterine sarcomas. Mol Clin Oncol. 2018; 8:499–503. https://doi.org/10.3892/mco.2018.1560.
15. Díaz-Montes TP, El-Sharkawy F, Lynam S, Harper A, Sittig M, MacDonald R, Gushchin V, Sardi A. Efficacy of Hyperthermic Intraperitoneal Chemotherapy and Cytoreductive Surgery in the Treatment of Recurrent Uterine Sarcoma. Int J Gynecol Cancer. 2018; 28:1130–37. https://doi.org/10.1097/IGC.0000000000001289.
16. Wu TI, Chang TC, Hsueh S, Hsu KH, Chou HH, Huang HJ, Lai CH. Prognostic factors and impact of adjuvant chemotherapy for uterine leiomyosarcoma. Gynecol Oncol. 2006; 100:166–72. https://doi.org/10.1016/j.ygyno.2005.08.010.
