# Supplementary material for: Role of surgery in gynaecological sarcomas
Source: Oncotarget. 2019 Apr 2;10(26):2561–75. doi: 10.18632/oncotarget.26803 (PMC6493462; doi:10.18632/oncotarget.26803)
Supplement: Supplementary file 2 [file oncotarget-10-2561-s002.docx]

**Supplementary Table 1: Overview of main studies on the role of surgery in uterine sarcoma**

| Authors | Year | Number of patients | Type of study | Type of sarcoma | Setting | FIGO Stage, N (%) | Risk of LN metastasis | Risk of ovarian metastasis* | Survival | Conclusion of the study | Note |
| --- | --- | --- | --- | --- | --- | --- | --- | --- | --- | --- | --- |
| Barney et al. [1] | 2009 | 1010 | Retrospective | ESS | Primary disease | I: 797 (78.9%)  II: 55 (5.4%)  III: 141 (14.0%)  IV: 17 (1.7%) | 2.1% (FIGO stage IIIC) | 0.7% (FIGO stage IIA) | 5-year OS (surgery alone) 83.2% | Worse prognoses were associated with increasing FIGO stage, tumor grade, and age. Neither did adjuvant RT correlate with improved survival within any FIGO stage nor did it alter survival for low- or high-grade tumor groups. Adding lymphadenectomy to TAH-BSO did not change survival. | National Cancer Institute’s Surveillance, Epidemiology, and End Results Program (1983-2002) |
| Seagle et al. [2] | 2017 | 7455 | Retrospective | uLMS | Primary disease | I: 1019 (13.7%)  II: 217 (2.9%)  III: 222 (3.0%)  IV: 630 (8.4%)  NR: 5367 (72.0%) | 2.5% (3.9% of cases did not report data on LN surgery) | 2.9% (FIGO stage II) | 5-year OS, according to stage:  I: 55.4% II: 32.6% III: 24.6% IV: 13.1% (9.8–17.4) NR: 42.6 (41.3–44.1) | Early and complete resection is the best-evidenced treatment for uterine leiomyosarcoma. Oophorectomy and lymphadenectomy may be safely omitted for clinically uterus-confined leiomyosarcoma. Chemotherapy increases survival of women with metastatic leiomyosarcoma. | National Cancer Institute’s Surveillance, Epidemiology, and End Results Program (1998-2013) |
| Kapp et al. [3] | 2008 | 1396 | Retrospective | uLMS | Primary disease | I: 951 (68.1%)  II: 43 (3.1%)  III: 99 (7.1%)  IV: 303 (21.7%) | 6.6% (75.0% of patients did not undergo lymphadenectomy) | 3.1% (FIGO stage II) | 5-year disease-specific survival 65.7% | Independent predictors of disease-specific survival in patients with uLMS  included age, race, stage, grade, and primary surgery. Oophorectomy was not found to have an independent impact on survival. | National Cancer Institute's Surveillance, Epidemiology, and End Results database (1988–2003) |
| Leitao et al. [4] | 2003 | 275 (145 included) | Prospective | uLMS | Primary | I/II: 98 (67.6%)  III/IV: 47 (32.4%) | 0% in FIGO stage I/II, 8.1% of the whole cohort (all LN+ had FIGO stage III/IV disease) | 3.9% of the whole cohort. 2.8% in FIGO stage I/II, 5.4% in FIGO stage III/IV | NR | The incidence of ovarian and lymph node metastases in uterine LMS is very low and is most commonly associated with extra-uterine disease. Lymph node dissection for uterine LMS should be reserved for patients with clinically suspicious nodes | Patients treated 1982-2001 |
| Stewart et al. [5] | 2018 | 112 | Retrospective | LG-ESS | Primary | I: 37 (52.0%)  II-IV: 22 (31.0%) (17% missing data) | 13.8% (32.1% underwent lymph node sampling) | Recurrences occurred in 42.8% of patients with median  PFS of 53 months and OS of 63 months | PFS was 38 vs 11 months for those who underwent BSO vs those who retained their ovaries (p=0.55) | BSO prolongs PFS in LG-ESS but these results are limited by sample size of the study | Patients treated 1985-2014 |
| Nasioudis et al. [6] | 2017 | 1482 | Retrospective | uLMS, LG-ESS, AS | Primary; FIGO stage I only | I: 1482 (100%) | NR | NR | 5-year cancer specific survival: ovarian preservation group: 84.8%; oophorectomy group: 82.9% | Ovarian preservation was not associated with worse oncologic outcomes. Ovarian preservation could be considered for women with uLMS, sparing them from the morbidity associated with iatrogenic menopause. No conclusions could be made for those with LG-ESS or AS. | National Cancer Institute's Surveillance, Epidemiology, and End Results database (1988-2013) |
| Shah et al. [7] | 2008 | 970 | Retrospective | ESS | Primary | LG-ESS: I: 254 (71.3%)  II: 13 (3.7%) III: 54 (15.2%) IV: 35 (9.8%)  NR: 28; HG-ESS: I: 97 (39.8%)  II: 19 (7.7%)  III: 47 (19.3%)  IV: 81 (33.2%)  NR: 76 | 7% (of the 26.1% LG-ESS patients who underwent lymphadenectomy); 18% (of the 45.3% HG-ESS patients who underwent lymphadenectomy) | Incidence of extra-uterine disease: 25% | 5-year OS LG-ESS according to stage: I/II: 96%, III/IV: 81% | In LG-ESS, the risk of extrauterine spread and lymph node metastasis merit consideration for surgical staging. Neither lymph node metastasis nor ovarian preservation seems to affect the excellent overall survival of these patients. | National Cancer Institute’s Surveillance, Epidemiology, and End Results Program (1988-2005) |
| Bai et al. [8] | 2014 | 153 | Retrospective | LG-ESS | Primary | I: 130 (85.0%)  II: 13 (8.5%)  III: 9 (5.9%)  IV: 1 (0.7%) | 2.7% (only 30.1% of patients underwent lymphadenectomy) | 5.9% patients with adnexal metastases | Mean follow-up time of 74.2 (range: 1–396) months: 32.0% recurred; 5.9% died of disease | Hysterectomy with bilateral salpingo-oophorectomy and complete resection of the macroscopic lesion should be treated as the initial and salvage mainstay treatments for LG-ESS patients. Ovary-sparing procedures could be considered for young women without cervical involvement; however, long-term follow-up should be mandatory. | Patients treated 1979-2013 |
| Lissoni et al. [9] | 1998 | 8 | Retrospective | uLMS | Fertility-sparing surgery | I: 8 (100%) | NA | NA | Median follow-up: 42 (range: 11-92) months: 1 patient died of disease, 7 patients alive without disease | Selected cases of uterine leiomyosarcoma might be managed conservatively in young nulliparous women desiring pregnancy. A strict follow-up is mandatory, and at the completion of the reproductive life, a demolitive procedure could be considered. | Patients treated 1982-1996 with myomectomy |
| Xie et al [10] | 2017 | 17 | Retrospective | LG-ESS | Fertility-sparing surgery | IA: 6 (35.3%)  IB: 11 (64.7%) | NA | NA | Median follow-up: 39 (range: 4-106) months: 10 (58.8%) recurred once, 3 (17.6%) had second recurrence, no patient died | Fertility-sparing surgery may be considered for young patients with stage IA low-grade ESS who wish to preserve their fertility. | Patients treated 2001-2015 with fertility-sparing surgery |
| Dinh et al [11] | 2004 | 27 | Retrospective | uLMS | Primary | I: 7 (25.9%)  II: 3 (11.1%)  III: 1 (3.7%)  IV: 16 (59.3%) | 0% (39% of patients underwent lymphadenectomy) | 11.1% (FIGO stage II) | 5-year OS: 42% (median follow-up 58 months) | Aggressive surgical cytoreduction at the time of initial diagnosis offers the possibility of prolonged survival or cure. | Patients treated 1990-1999 |
| Leitao et al [12] | 2012 | 96 | Retrospective | uLMS | Metastatic/Advanced stage | Metastatic: intra-peritoneal 48 (50%), extra-peritoneal 48 (50%) | NR | NR | Median PFS: 8.7 (6.7-10.9) months; Median OS: 20.2 (15.5-24.8) months | Surgical cytoreduction of metastatic uterine LMS was independently associated with PFS but not OS in cases selected for surgery. The improvement in PFS must be weighed against the morbidity of surgery. | Patients treated 1982-2007 |
| Giuntoli et al. [13] | 2007 | 128 | Retrospective | uLMS | Recurrent | I: 82 (64%)  II: 10 (8%)  III: 13 (10%)  IV: 17 (13%) | NR | NR | Median DFS: 1.3 years, median DSS: 3.3 years, median DSS (from 1^st^ recurrence): 1.8 years | Secondary cytoreductive surgery is associated with prolonged survival in a select group of patients with recurrent uterine LMS. Patients presenting after a prolonged progression-free interval with an isolated site of recurrence amenable to complete resection are the best candidates for attempted surgical resection. | Patients treated 1976-1999 at multiple institutions |
| Nakamura et al. [14] | 2018 | 18 | Retrospective | 8 uLMS  6 carcinosarcoma  3 ESS  1 AS | Recurrent | I: 9 (50%)  II: 0  III: 6 (33.3%)  IV: 3 (16.7%) | NR | NR | 3‑year OS were 77.8% and 11.1% in the SCS and non‑SCS groups | Resection of recurrent uterine sarcomas may be beneficial for the improvement of patient survival | Patients treated 2002-2015 |
| Díaz-Montes et al [15] | 2018 | 26 | Retrospective | 22 uLMS  2 ESS  2AS | Recurrent | I: 9 (34.6%)  II: 5 (19.2%)  III: 4 (15.4%)  IV: 6 (23.1%)  NR: 2 | NR | NR | Median OS: 35.9 months for conventional surgery, 43.8 months for HIPEC | Adding HIPEC to conventional therapies is beneficial in recurrent uterine sarcoma | Patients treated 2005-2016 |
| Wu et al. [16] | 2006 | 51 | Retrospective | uLMS | Primary | I: 41 (80.4%)  II: 0  III: 7 (13.7%)  IV: 3 (5.9%) | 0/21 patients who underwetn lymphadenectomy | NR | Median follow-up for survivors was 47 months. 5-year OS and RFS were 67.4% and 59.2% | Benefit of adjuvant chemotherapy for uLMS | Patients treated 1984-2003 |

LN=lymph nodes, ESS=endometrial stroma sarcoma, uLMS=uterine leiomyosarcoma, NR=not reported, OS=overall survival, LG=low grade, HG=high grade, BSO=bilateral salpingo-oophorectomy, PFS=progression-free survival, AS=adenosarcoma, NA=not applicable, DFS=disease-free survival, DSS=disease specific survival, RFS=recurrence-free survival

* This data could be underestimated as patients with FIGO stage worse than II could have had ovarian metastasis, but this was not reported in the original papers.
